# Supplementary material for: Genome-Wide Identification of Epigenetic Regulators in Quercus suber L
Source: Int J Mol Sci. 2020 May 27;21(11):3783. doi: 10.3390/ijms21113783 (PMC7313042; doi:10.3390/ijms21113783)
Supplement: Supplementary file 1 [file ijms-21-03783-s001.pdf]

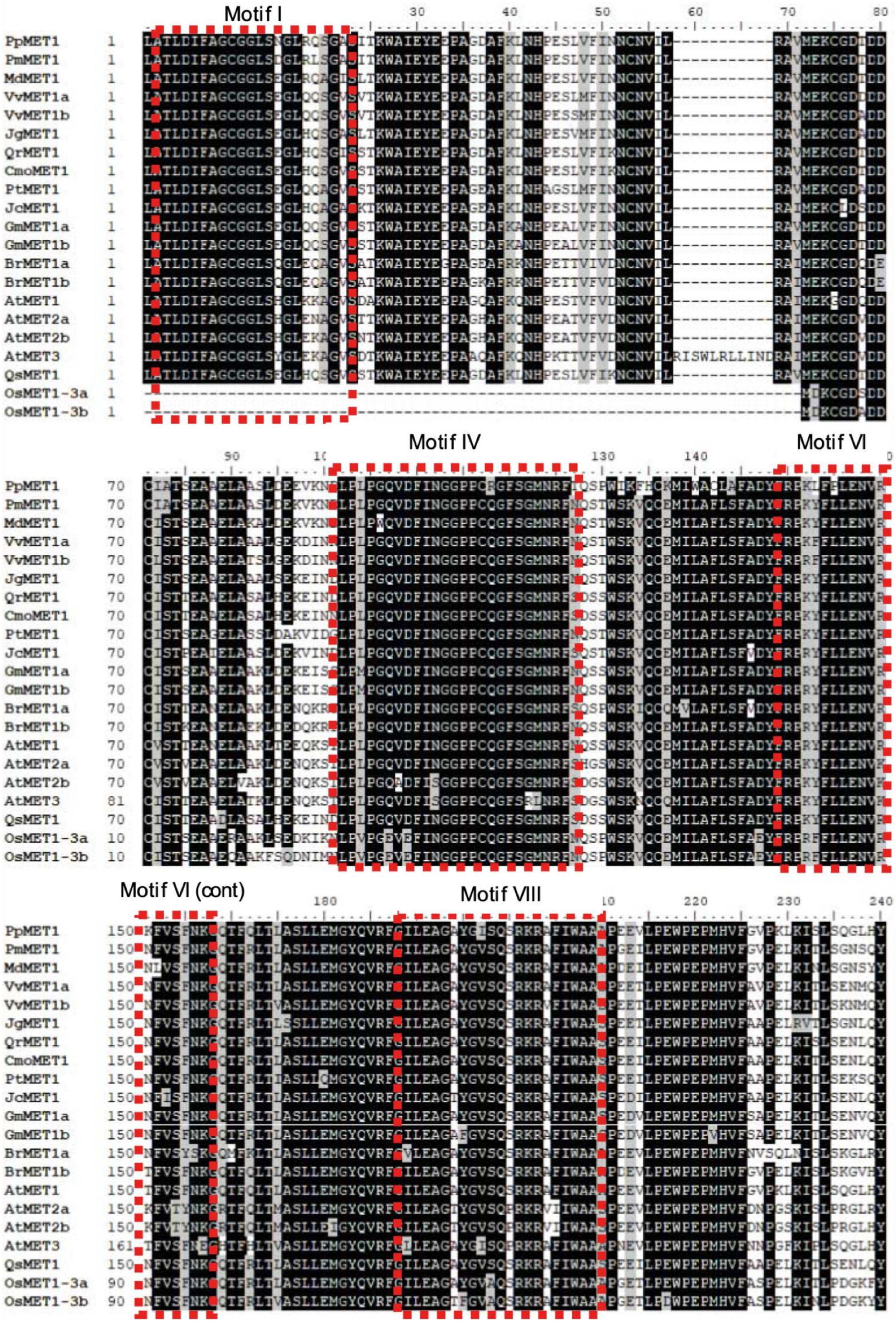
Motif I
Motif IV
Motif VI
Motif VI (cont)
Motif VIII

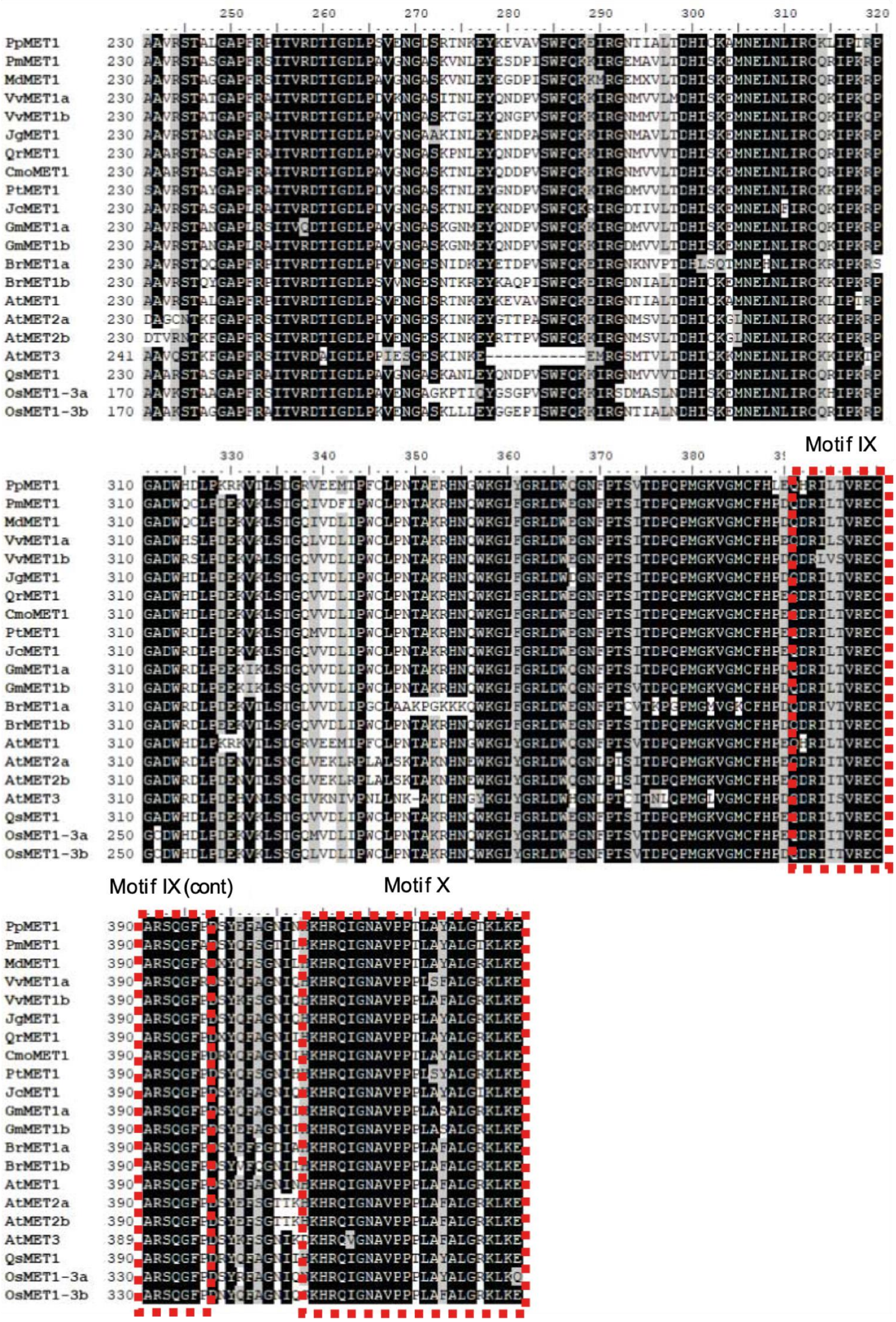

Supplementary Figure S1. Alignment of the amino acid sequences of MET family proteins. Dotted red boxes indicate the conserved methyltransferase catalytic motifs I, IV, and VI, VIII-X. Black background shows identical amino acid sequences among MET1 proteins.

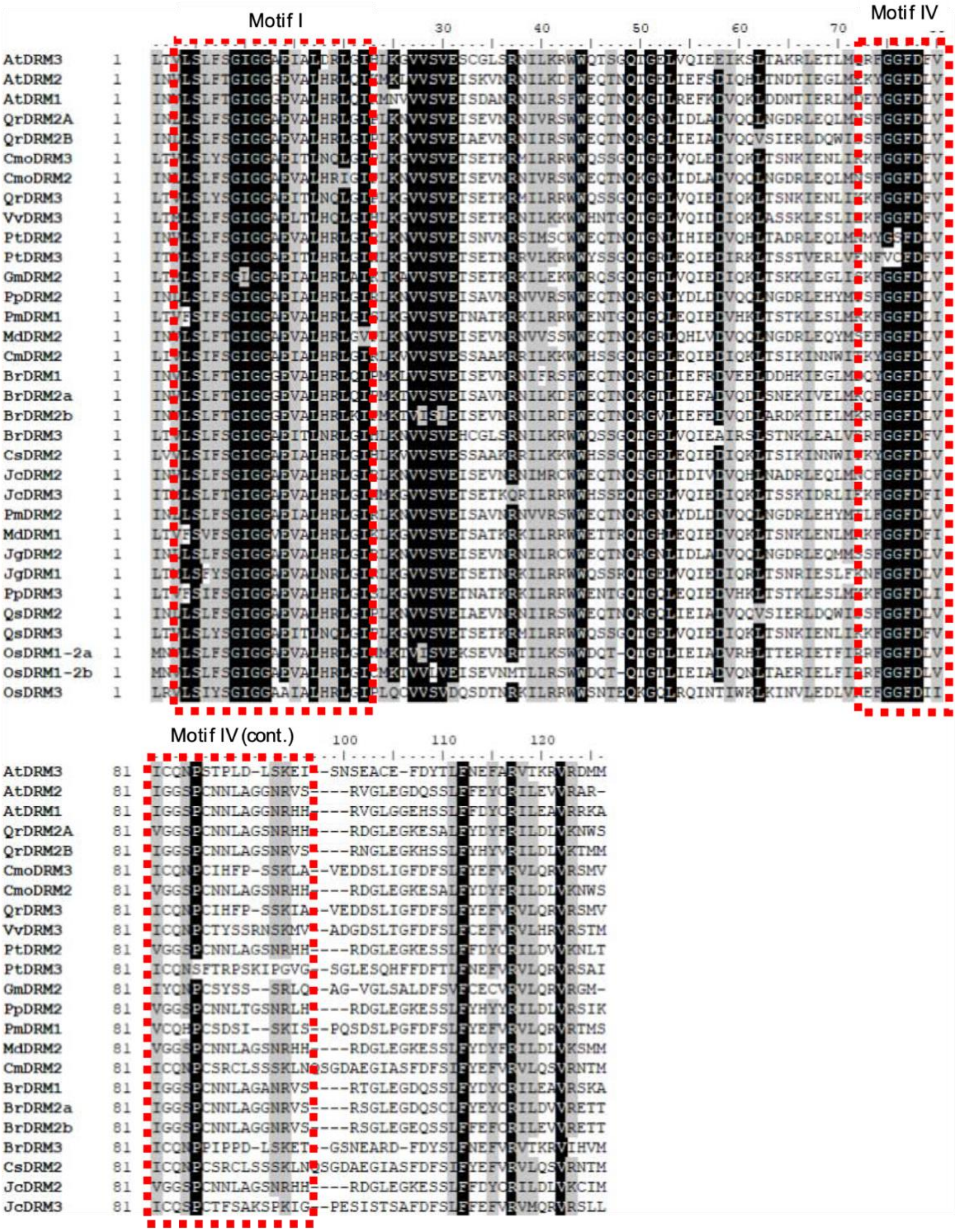

Supplementary Figure S2. Alignment of the amino acid sequences of DRM family proteins. Dotted red boxes indicate the conserved methyltransferase catalytic motifs I, and IV. Black background shows identical amino acid sequences among DRM proteins.

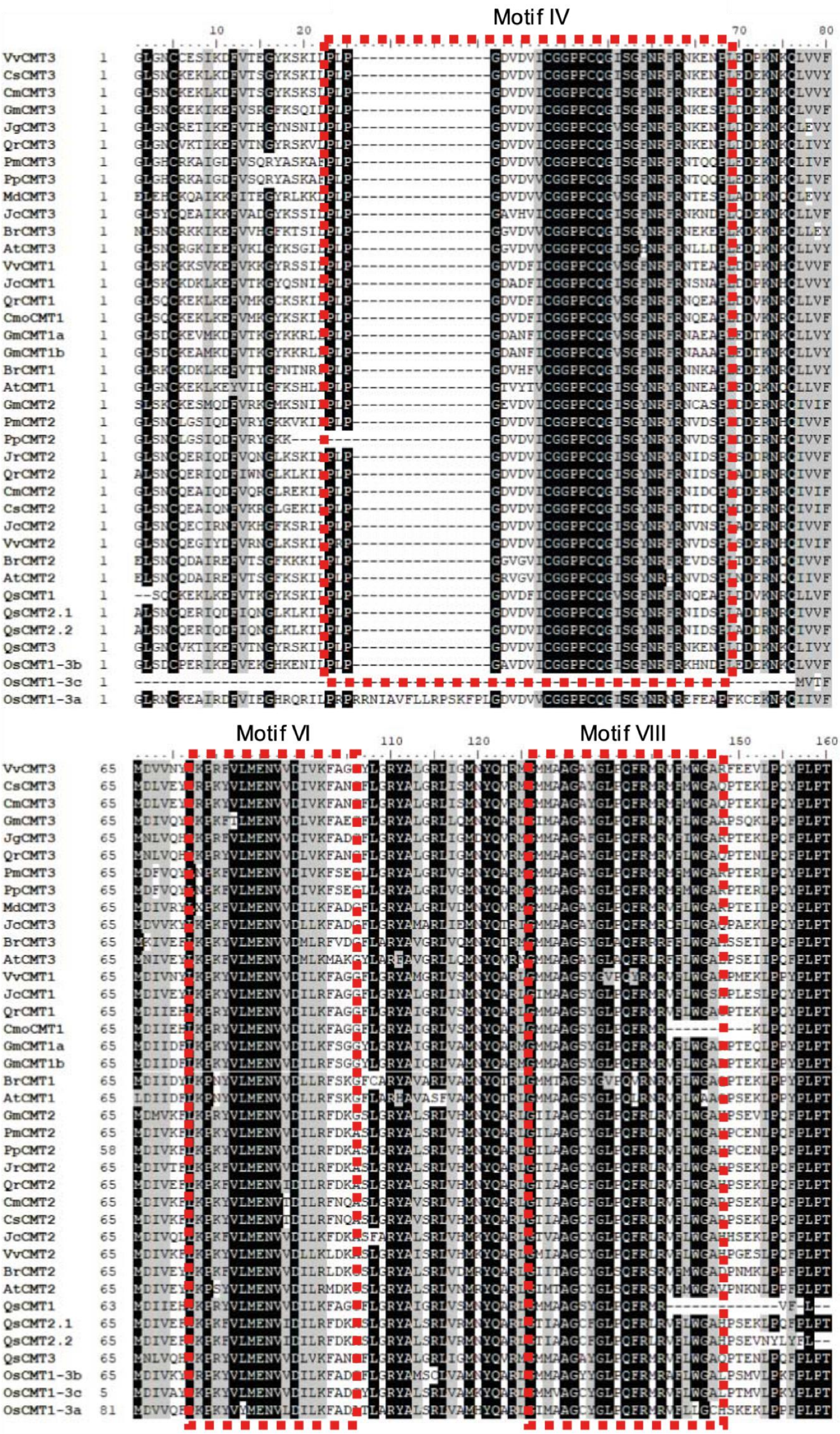

Motif IV
Motif VI
Motif VIII
VvCMT3
CsCMT3
CmCMT3
GmCMT3
JgCMT3
QrCMT3
PmCMT3
PpCMT3
MdCMT3
JcCMT3
BrCMT3
AtCMT3
VvCMT1
JcCMT1
QrCMT1
CmoCMT1
GmCMT1a
GmCMT1b
BrCMT1
AtCMT1
GmCMT2
PmCMT2
PpCMT2
JrCMT2
QrCMT2
CmCMT2
CsCMT2
JcCMT2
VvCMT2
BrCMT2
AtCMT2
QsCMT1
QsCMT2.1
QsCMT2.2
QsCMT3
OsCMT1-3b
OsCMT1-3c
OsCMT1-3a

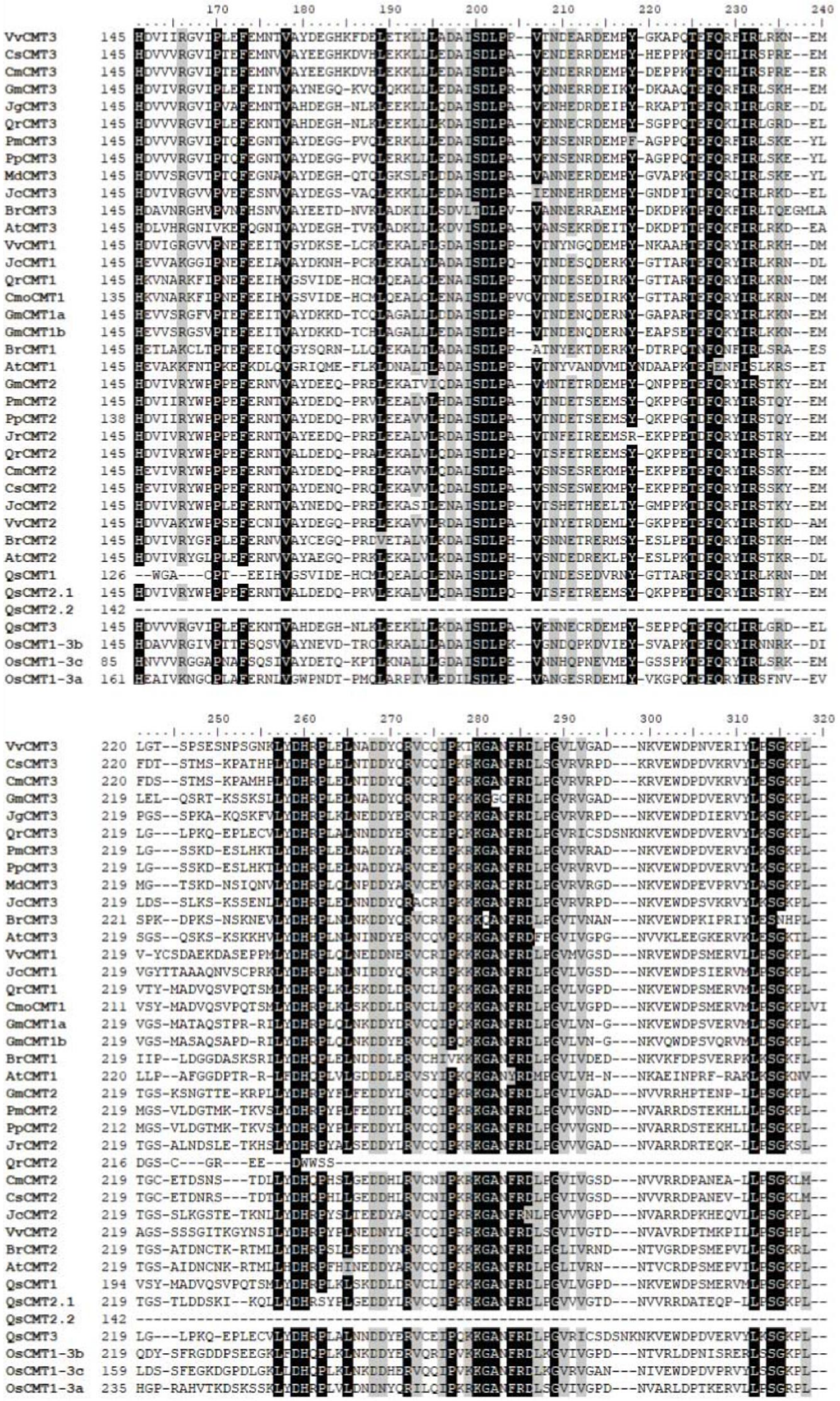

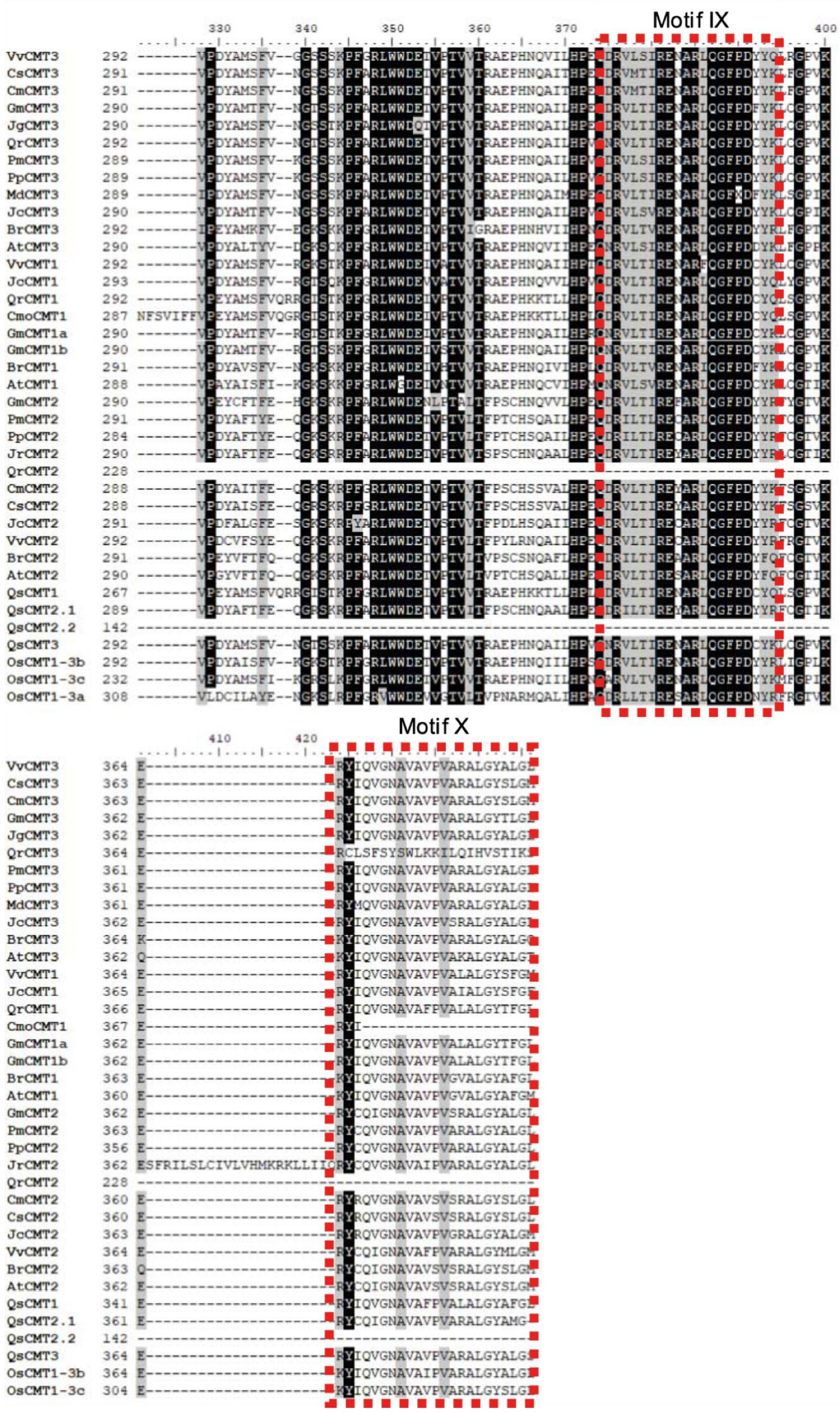

Supplementary Figure S3. Alignment of the amino acid sequences of CMT family proteins. Dotted red boxes indicate the conserved methyltransferase catalytic motifs IV, VI and VIII-X. Black background shows identical amino acid sequences among CMT proteins.

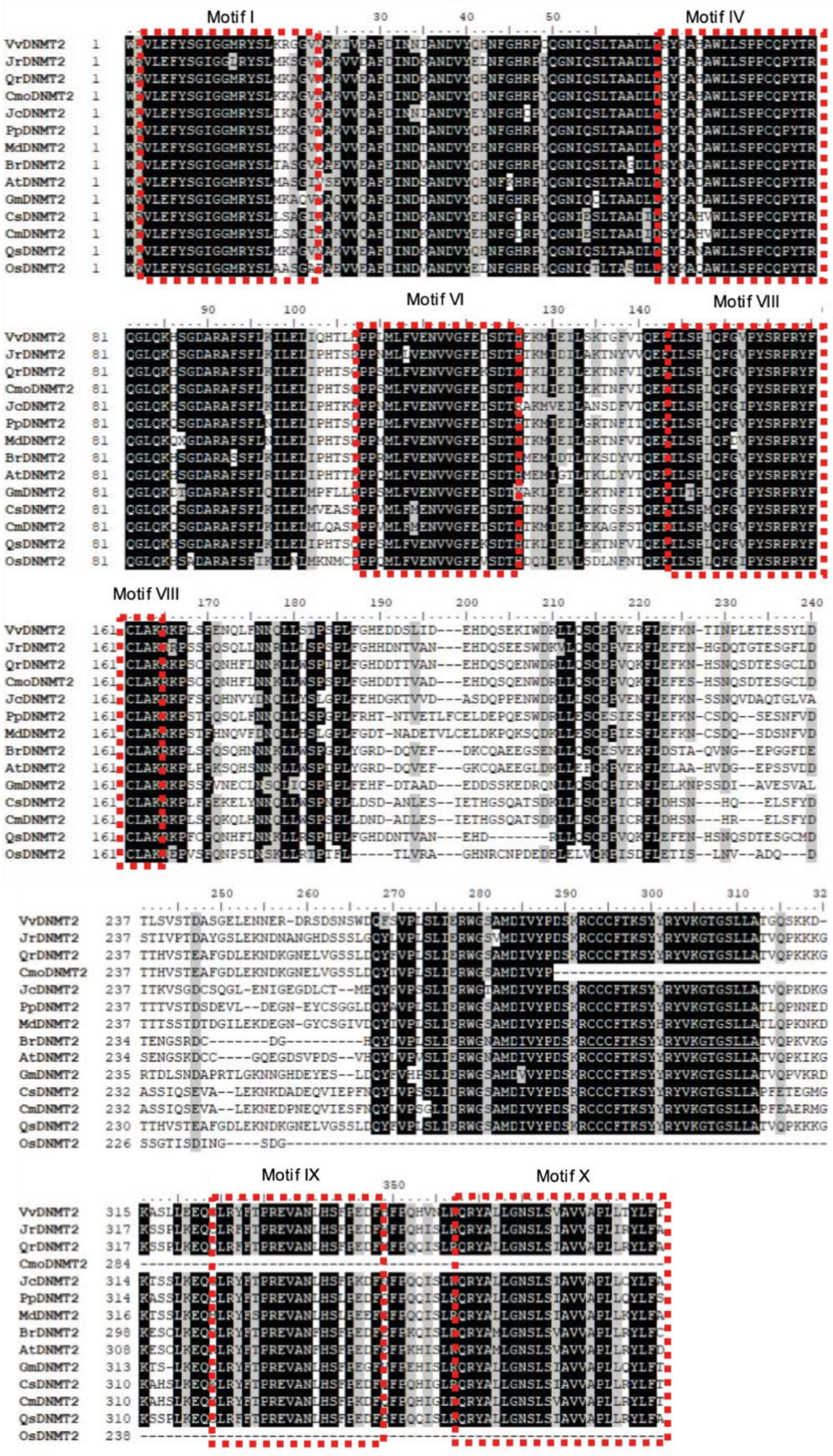

Supplementary Figure S4. Alignment of the amino acid sequences of DNMT family proteins. Dotted red boxes indicate the conserved methyltransferase catalytic motifs I, IV, VI and VIII-X. Black background shows identical amino acid sequences among DNMT proteins.

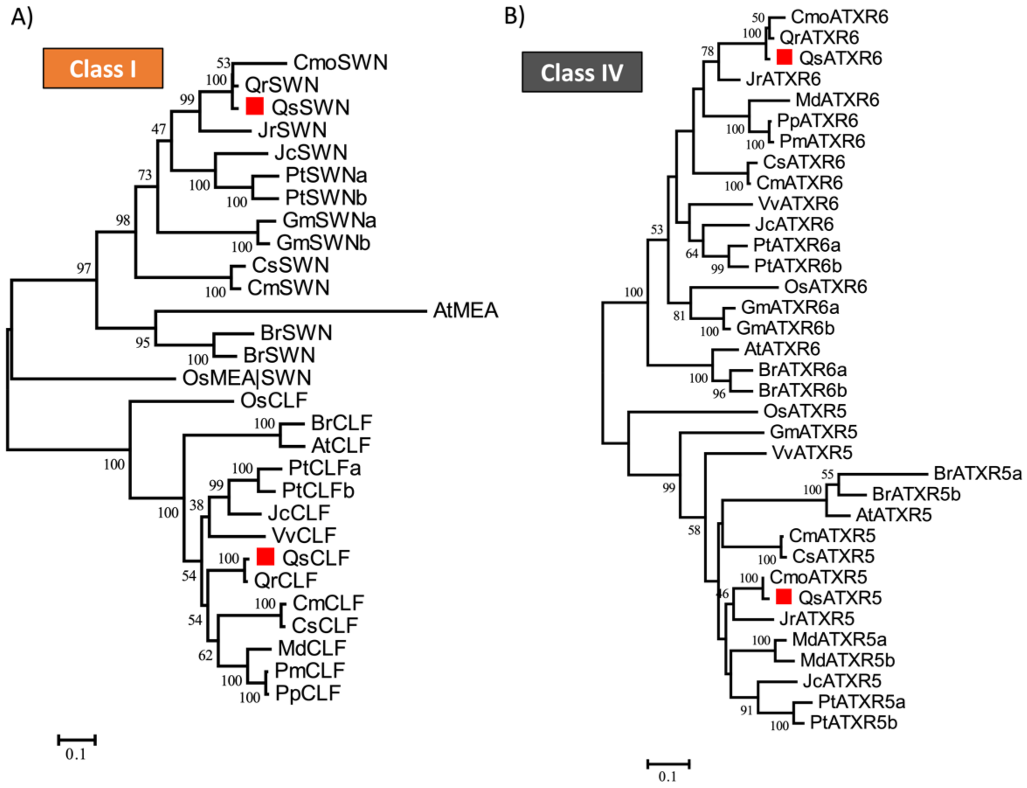

Supplementary Figure S5. Phylogeny of HMT proteins of class I and IV. The SET domain *Quercus suber L.* (Qs), *Arabidopsis thaliana* (L.) Heynh. (At), *Brassica rapa L.* (Br), *Glycine max* (L.) Merr. (Gm), *Jatropha curcas L.* (Jc), *Populus trichocarpa* Torr. & A.Gray ex Hook. (Pt), *Prunus persica* (L.) Batsch (Pp), *Prunus mume* (Siebold) Siebold & Zucc. (Pm), *Vitis vinifera L.* (Vv), *Cucumis melo L.* (Cm), *Cucumis sativus L.* (Cs), *Juglans regia* L. (Jr), *Castanea mollissima* Blume (Cmo), *Quercus robur L.* (Qr) and *Oryza sativa L.* (Os) were aligned using ClustalW and used to infer the evolutionary history using the Maximum-likelihood method. The evolutionary distances (left side scale bar) were computed using the Jones-Taylor-Thornton (JTT) correction model. The numbers at the nodes represent bootstrap values from 1000 replicates. The *Q. suber* SET domain contain proteins were indicated with a red square. Phylogenetic analyses were conducted in MEGA7.

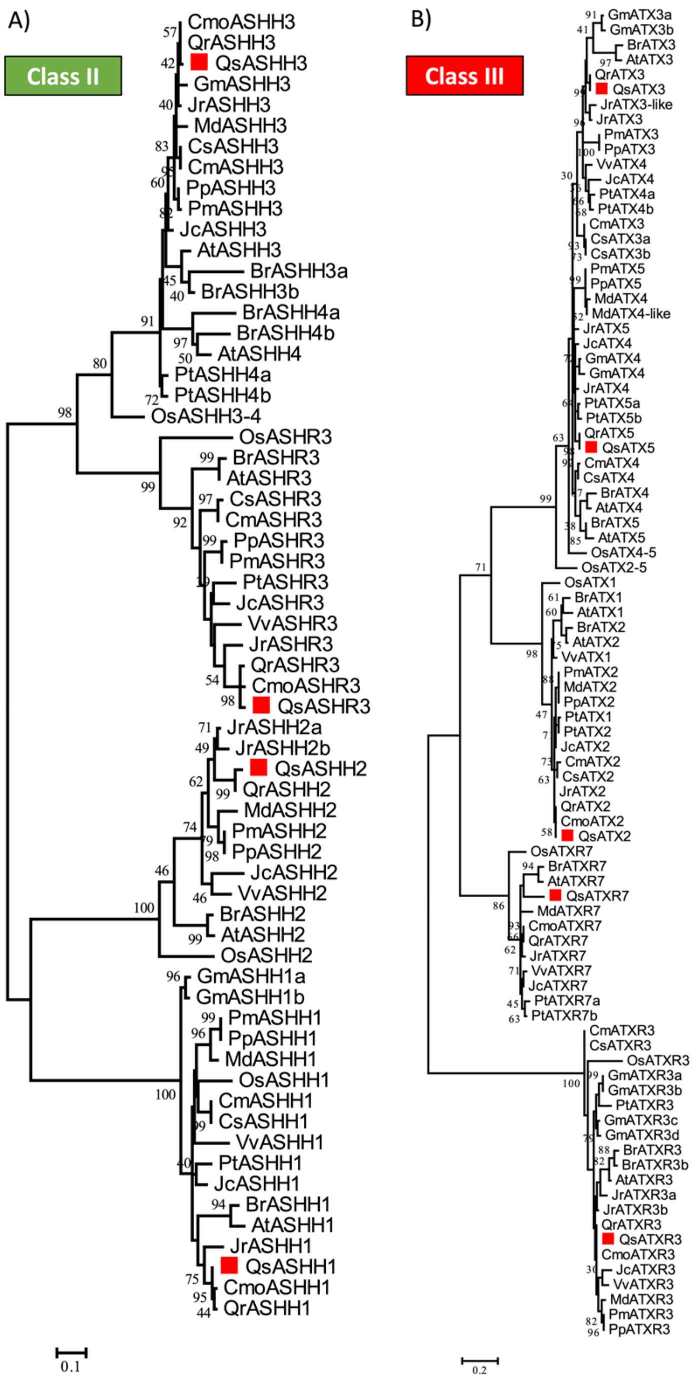

Supplementary Figure S6. Phylogeny of HMT proteins of class II and III. The SET domain of *Quercus suber* L. (Qs), *Arabidopsis thaliana* (L.) Heynh. (At), *Brassica rapa* L. (Br), *Glycine max* (L.) Merr. (Gm), *Jatropha curcas* L. (Jc), *Populus trichocarpa* Torr. & A.Gray ex Hook. (Pt), *Prunus persica* (L.) Batsch (Pp), *Prunus mume* (Siebold) Siebold & Zucc. (Pm), *Vitis vinifera* L. (Vv), *Cucumis melo* L. (Cm), *Cucumis sativ*us L. (Cs), *Juglans regia* L. (Jr), *Castanea mollissima* Blume (Cmo), *Quercus robur* L. (Qr) and *Oryza sativa* L. (Os) were aligned using ClustalW and used to infer the evolutionary history using the Maximum-likelihood method. The evolutionary distances (left side scale bar) were computed using the Jones-Taylor-Thornton (JTT) correction model. The numbers at the nodes represent bootstrap values from 1000 replicates. The *Q. suber* SET domain contain proteins were indicated with a red square. Phylogenetic analyses were conducted in MEGA7.

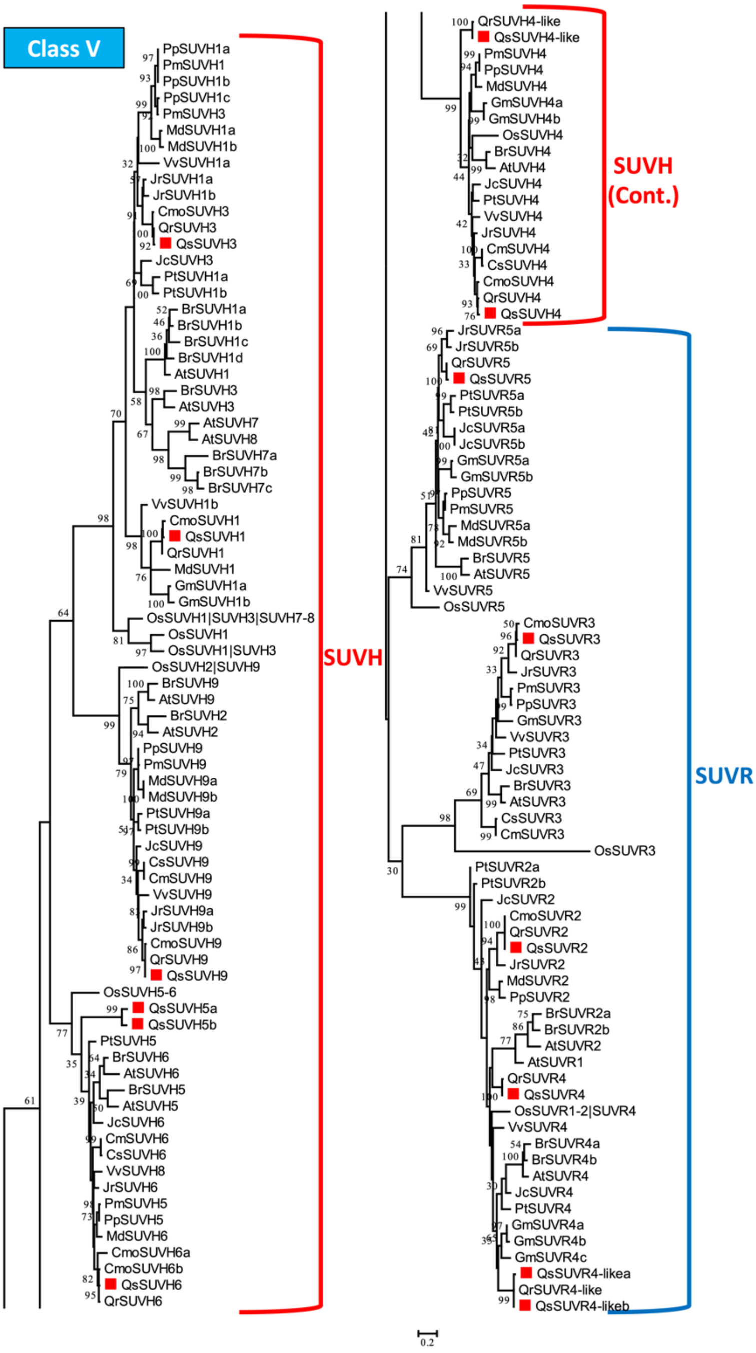

Supplementary Figure S7. Phylogeny of HMT proteins of class V. The SET domain of *Quercus suber* L. (Qs), *Arabidopsis thaliana* (L.) Heynh. (At), *Brassica rapa* L. (Br), *Glycine max* (L.) Merr. (Gm), *Jatropha curcas* L. (Jc), *Populus trichocarpa* Torr. & A.Gray ex Hook. (Pt), *Prunus persica* (L.) Batsch (Pp), *Prunus mume* (Siebold) Siebold & Zucc. (Pm), *Vitis vinifera* L. (Vv), *Cucumis melo* L. (Cm), *Cucumis sativus* L. (Cs), *Juglans regia* L. (Jr), *Castanea mollissima* Blume (Cmo), *Quercus robur* L. (Qr) and *Oryza sativa* L. (Os) were aligned using ClustalW and used to infer the evolutionary history using the Maximum-likelihood method. The evolutionary distances (left side scale bar) were computed using the Jones-Taylor-Thornton (JTT) correction model. The numbers at the nodes represent bootstrap values from 1000 replicates. The *Q. suber* SET domain contain proteins were indicated with a red square. Phylogenetic analyses were conducted in MEGA7.

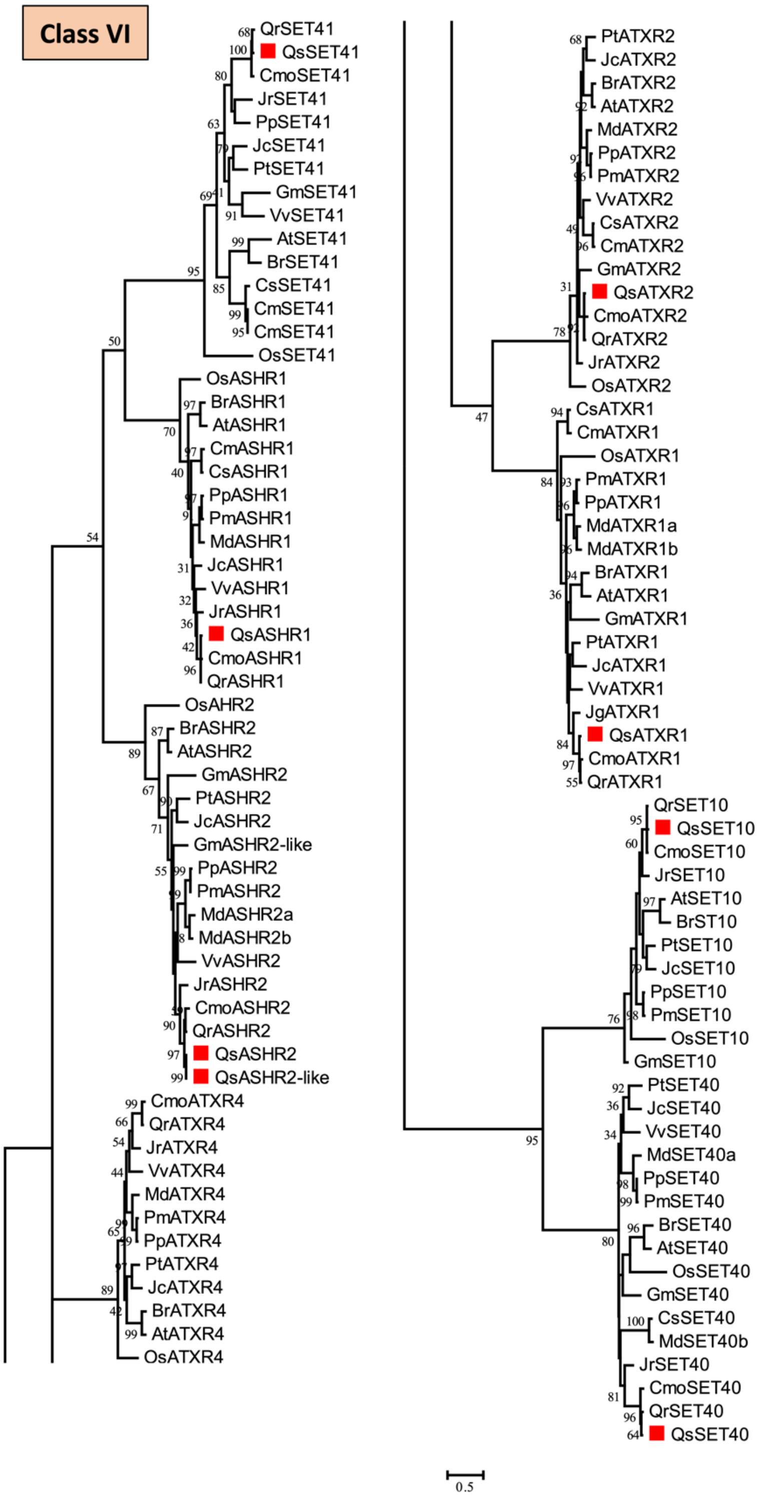

Supplementary Figure S8. Phylogeny of other SET containing proteins. The SET domain of *Quercus suber L.* (Qs), *Arabidopsis thaliana* (L.) Heynh. (At), *Brassica rapa* L. (Br), *Glycine max* (L.) Merr. (Gm), *Jatropha curcas* L. (Jc), *Populus trichocarpa* Torr. & A.Gray ex Hook. (Pt), *Prunus persica* (L.) Batsch (Pp), *Prunus mume* (Siebold) Siebold & Zucc. (Pm), *Vitis vinifera* L. (Vv), *Cucumis melo* L. (Cm), *Cucumis sativus* L. (Cs), *Juglans* regia L. (Jr), *Castanea mollissima* Blume (Cmo), *Quercus robur* L. (Qr) and *Oryza sativa* L. (Os) were aligned using ClustalW and used to infer the evolutionary history using the Maximum-likelihood method. The evolutionary distances (left side scale bar) were computed using the Jones-Taylor-Thornton (JTT) correction model. The numbers at the nodes represent bootstrap values from 1000 replicates. The *Q. suber* SET domain contain proteins were indicated with a red square. Phylogenetic analyses were conducted in MEGA7.

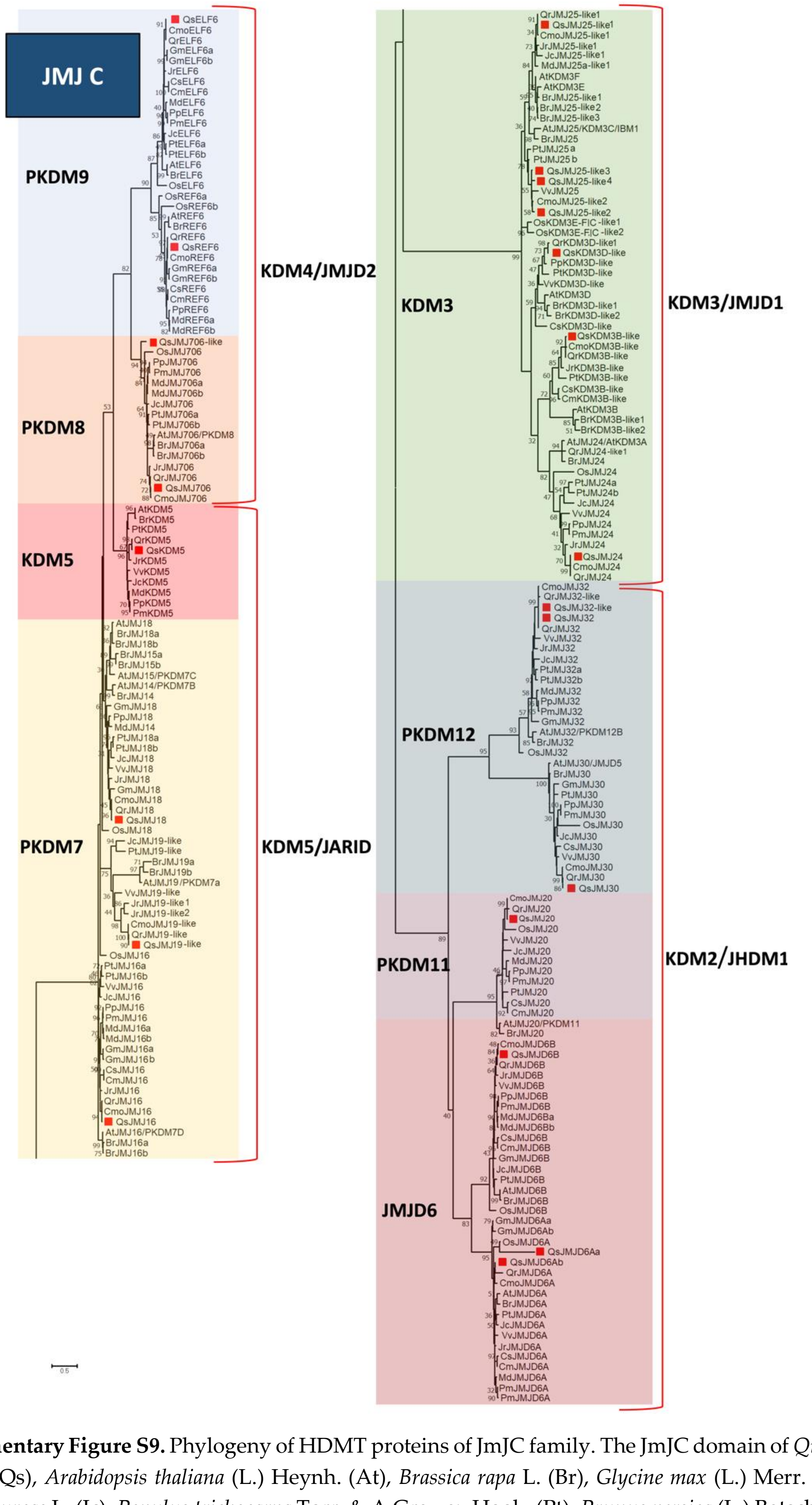

Supplementary Figure S9. Phylogeny of HDMT proteins of JmJC family. The JmJC domain of *Quercus suber* L. (Qs), *Arabidopsis thaliana* (L.) Heynh. (At), *Brassica rapa* L. (Br), *Glycine max* (L.) Merr. (Gm), *Jatropha curcas* L. (Jc), *Populus trichocarpa* Torr. & A.Gray ex Hook. (Pt), *Prunus persica* (L.) Batsch (Pp), *Prunus mume* (Siebold) Siebold & Zucc. (Pm), *Vitis vinifera* L. (Vv), *Cucumis melo* L. (Cm), *Cucumis sativus* L. (Cs), *Juglans regia* L. (Jr), *Castanea mollissima* Blume (Cmo), *Quercus robur* L. (Qr) and *Oryza sativa* L. (Os) were aligned using ClustalW and used to infer the evolutionary history using the Maximum-likelihood method. The evolutionary distances (left side scale bar) were computed using the Jones-Taylor-Thornton (JTT) correction model. The numbers at the nodes represent bootstrap

values from 1000 replicates. The *Q. suber* proteins were indicated with a red square. Phylogenetic analyses were conducted in MEGA7.

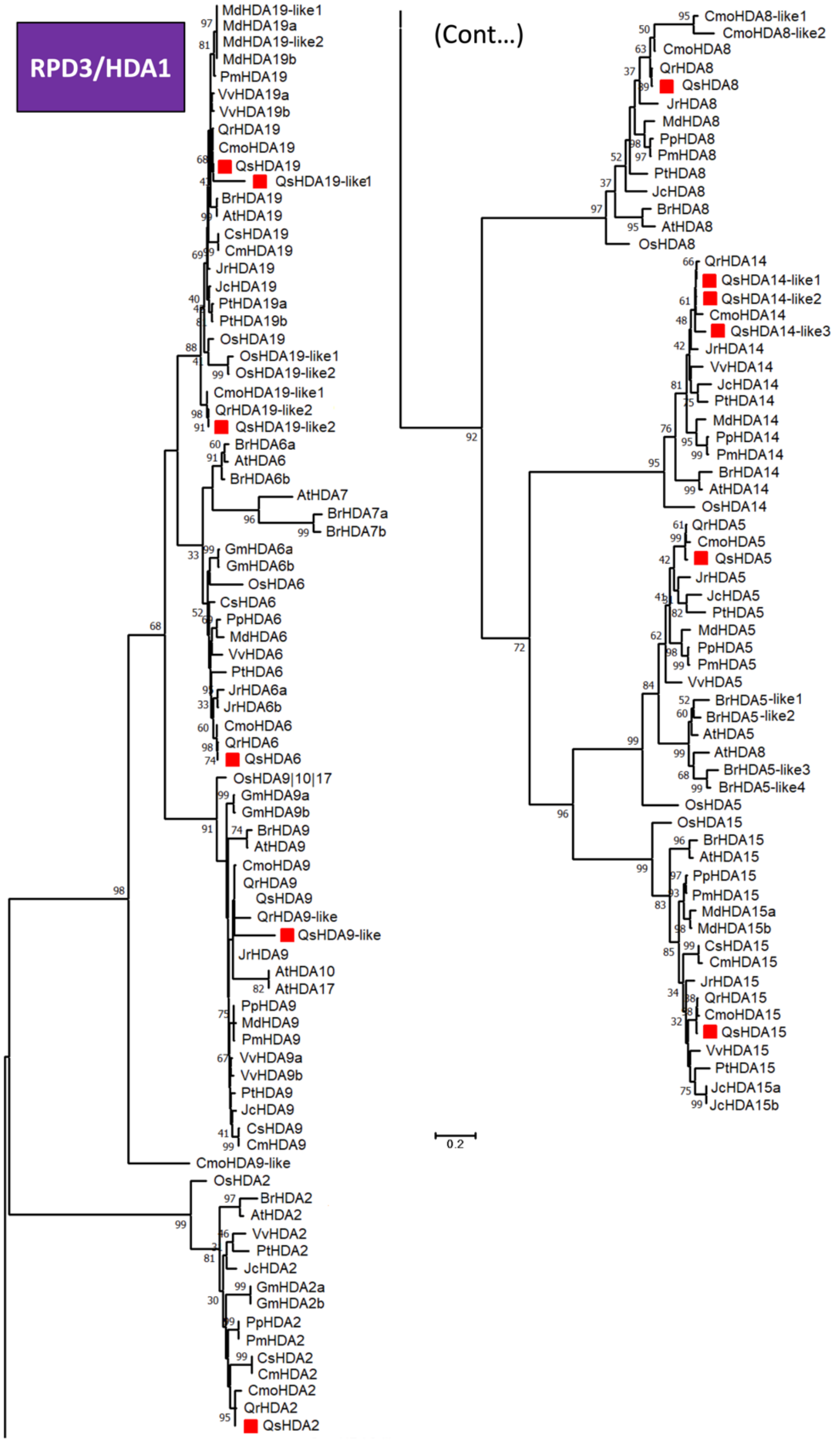

Supplementary Figure S10. Phylogeny of HDAC proteins of RPD3/HDA1 family. The HDAC domain of *Quercus suber* L. (Qs), *Arabidopsis thaliana* (L.) Heynh. (At), *Brassica rapa* L. (Br), *Glycine max* (L.) Merr. (Gm), *Jatropha curcas* L. (Jc), *Populus trichocarpa* Torr. & A.Gray ex Hook. (Pt), *Prunus persica* (L.) Batsch (Pp), *Prunus mume* (Siebold) Siebold & Zucc. (Pm), *Vitis vinifera* L. (Vv), *Cucumis melo* L. (Cm), *Cucumis sativus* L. (Cs), *Juglans regia* L. (Jr), *Castanea mollissima* Blume (Cmo), *Quercus robur* L. (Qr) and *Oryza sativa* L. (Os) were aligned using ClustalW and used to infer the evolutionary history using the Maximum-likelihood method. The evolutionary distances (left side scale bar) were computed using

the Jones-Taylor-Thornton (JTT) correction model. The numbers at the nodes represent bootstrap values from 1000 replicates. The *Q. suber* proteins were indicated with a red square. Phylogenetic analyses were conducted in MEGA7.

Table S1. Normalized read counts of DNA De/methyltransferases and histone modifiers detected in ecodormant bud (ecodB), swelling bud (swB), differentiating secondary xylem (XY), root (RO), leaf (LE) and *in vitro* dedifferentiated *callus* (CA) of *Quercus robur* [53].

Gene Name	Gene ID	EcoDB	swB	XY	RO	LE	CA
**DNA methyltransferases**							
QrCMT3	Loc_5202_Tr_1/2_Conf_0.889_Len_3208	0.0	44.9	0.8	3.1	7.8	4.1
QrCMT1	Loc_21057_Tr_1/5_Conf_0.667_Len_2822	7.7	185.6	5.9	96.7	45.6	54.0
QrCMT2	OCV4_rep_c4395	ND	ND	ND	ND	ND	ND
QrMET1	OCV4_c25957	874.6	2363.3	811.8	819.9	717.4	828.9
QrDNMT2	OCV4_rep_c6323/Loc_8252_Tr_6/6_Conf_0.600_Len_1795	1.9	0.8	2.5	3.1	1.3	1.0
QrDRM2A	Loc_1382_Tr_6/6_Conf_0.667_Len_2740	8.7	20.8	16.0	9.3	9.1	15.3
QrDRM2B	Loc_82954_Tr_2/2_Conf_0.750_Len_1158	1.9	0.0	3.4	0.0	0.0	0.0
QrDRM3	OCV4_rep_c18566	ND	ND	ND	ND	ND	ND
**Histone acetyltransferases**							
QrHAC1	OCV4_rep_c42029	ND	ND	ND	ND	ND	ND
QrHAC1-like2	Loc_39724_Tr_1/2_Conf_0.750_Len_4836	259.5	61.6	174.7	143.0	148.4	24.4
QrHAM1	OCV3_prime_c11529	366.2	435.2	232.1	524.7	623.6	299.4
QrHAG2	OCV4_rep_c26572	ND	ND	ND	ND	ND	ND
QrELP3	Loc_4308_Tr_2/4_Conf_0.600_Len_2295	74.0	59.9	123.2	94.6	74.2	279.0
QrGCN5	OCV4_c7575	938.1	1235.8	966.2	825.1	757.7	1486.7
QrHAF1	OCV4_rep_c6162	ND	ND	ND	ND	ND	ND
**Histone methyltransferases**							
QrSWN	Loc_1192_Tr_4/8_Conf_0.696_Len_3240	26.0	10.8	27.0	18.5	13.0	26.5
QrCLF	OCV4_rep_c16251/Loc_19971_Tr_7/10_Conf_0.467_Len_2918	16.3	24.1	12.7	14.4	5.2	6.1
QrASHH1	Loc_12859_Tr_1/4_Conf_0.769_Len_2491	36.5	20.8	30.4	26.7	7.8	12.2
QrASHH3	OCV4_rep_c40652/OCV4_rep_c14852	83.6	66.6	75.1	67.9	72.9	116.1
QrASHR3	OCV4_rep_c19155	ND	ND	ND	ND	ND	ND
QrASHH2	OCV4_rep_c21713	ND	ND	ND	ND	ND	ND
QrATXR7	Loc_9902_Tr_7/8_Conf_0.682_Len_7488	100.0	34.1	61.6	39.1	57.3	73.3
QrATX3	Loc_20589_Tr_3/6_Conf_0.688_Len_3877	936.2	912.9	813.5	788.0	1070.2	643.6

QrATX5	OCV4_c26314	353.7	290.4	505.5	259.2	251.3	242.4
QrATX2	OCV4_rep_c43523	ND	ND	ND	ND	ND	ND
QrATXR3	OCV4_rep_c4016	ND	ND	ND	ND	ND	ND
QrATXR6	OCV4_rep_c10294	0.0	94.9	0.8	11.3	29.9	8.1
QrSUVR3	OCV4_rep_c24922	ND	ND	ND	ND	ND	ND
QsSUVH6	OCV4_c18	894.8	1056.0	1145.1	667.7	820.2	809.5
QrSUVH4	OCV4_rep_c40958/OCV4_rep_c14813	380.6	687.4	457.4	475.3	407.5	280.0
QrSUVH4-like	Loc_19624_Tr_8/9_Conf_0.609_Len_2652	34.6	51.6	34.6	32.9	52.1	44.8
QrSUVH1	OCV4_c19481	1715.7	1368.1	1861.6	1154.3	1394.4	2238.2
QrSUVH3	OCV4_rep_c21535	ND	ND	ND	ND	ND	ND
QrSUVH9	Loc_7424_Tr_3/4_Conf_0.545_Len_5241	75.9	37.4	88.6	38.1	20.8	101.8
QrSUVR4-like	OCV4_c20491	6.7	9.2	15.2	32.9	10.4	4.1
QrSUVR4	Loc_53227_Tr_1/1_Conf_1.000_Len_2710	50.0	45.8	0.8	22.6	6.5	4.1
QrSUVR2	OCV4_rep_c16379	ND	ND	ND	ND	ND	ND
QrSUVR5	Loc_7632_Tr_2/3_Conf_0.800_Len_7750	127.8	164.8	161.2	123.5	89.8	334.0
QrSET40	Loc_19922_Tr_2/5_Conf_0.636_Len_1707	336.4	374.5	351.9	153.3	149.7	291.2
QrSET10	Loc_11855_Tr_5/6_Conf_0.611_Len_2331	120.1	89.9	122.4	71.0	80.7	97.8
QrSET41	OCV4_c22093	321.0	461.8	378.9	452.7	416.6	356.4
QrASHR2	OCV4_rep_c11271	556.5	1317.3	582.3	935.1	565.1	635.4
QrASHR1	OCV4_rep_c3041	ND	ND	ND	ND	ND	ND
QrASHR1	OCV4_rep_c943/Loc_12215_Tr_7/9_Conf_0.583_Len_3513	41.3	20.0	34.6	26.7	20.8	42.8
QrATXR4	Loc_30882_Tr_3/3_Conf_0.714_Len_832	0.0	2.5	0.8	13.4	6.5	0.0
QrATXR1	Loc_33752_Tr_1/1_Conf_1.000_Len_2058	2.9	126.5	1.7	85.4	61.2	51.9
**Histone demethyltransferases**							
QrJMJ24	OCV4_c26318	674.7	501.8	426.2	310.7	665.3	506.1
QrJMJ25-like1	OCV4_c27580	68.2	167.3	54.9	204.7	112.0	118.1
QrKDM3D-like2	OCV4_rep_c9279	ND	ND	ND	ND	ND	ND
QrKDM3D-like1	OCV4_rep_c15058	483.5	1126.7	458.2	412.5	266.9	290.2
QrKDM3B-like	Loc_11265_Tr_3/5_Conf_0.692_Len_5052	19.2	67.4	19.4	8.2	14.3	22.4
QrJMJD6B	OCV4_rep_c21199	ND	ND	ND	ND	ND	ND

QrJMJD6A	OCV4_rep_c28281	ND	ND	ND	ND	ND	ND
QrJMJ32	Loc_19662_Tr_4/5_Conf_0.500_Len_1503	20.2	16.6	25.3	21.6	10.4	23.4
QrJMJ32-like	OCV4_rep_c1929	ND	ND	ND	ND	ND	ND
QrJMJ20	OCV4_rep_c11681	19.2	13.3	23.6	26.7	43.0	15.3
QrJMJ16	Loc_970_Tr_1/3_Conf_0.727_Len_4447	167.2	150.6	344.3	309.7	157.5	263.7
QrJMJ19-like	OCV4_rep_c18633	ND	ND	ND	ND	ND	ND
QrKDM5	Loc_1909_Tr_59/61_Conf_0.057_Len_4367	36.5	18.3	31.2	12.3	19.5	36.7
QrJMJ18	Loc_9658_Tr_3/4_Conf_0.700_Len_4718	11.5	28.3	18.6	19.5	24.7	35.6
QrREF6	OCV4_c5323	1482.1	3231.3	2221.1	1945.4	1809.7	1652.7
QrJMJ706	Loc_2899_Tr_3/5_Conf_0.417_Len_2953	79.8	163.9	60.8	451.6	287.7	36.7
QrELF6	OCV4_rep_c3275	ND	ND	ND	ND	ND	ND
QrLDL1	OCV4_rep_c28326	ND	ND	ND	ND	ND	ND
QrFLD	OCV4_rep_c42703	ND	ND	ND	ND	ND	ND
QrLDL2	OCV4_c28695	706.4	877.9	418.6	445.5	497.4	478.6
QrLDL3	Loc_2373_Tr_8/10_Conf_0.706_Len_11006	428.7	561.7	449.8	699.6	841.1	717.9

Histone deacetyltransferases

Gene name	**Gene ID**	**EcoDB**	**swB**	**XY**	**RO**	**LE**	**CA**
QrHDA19-like1	OCV4_rep_c21849	ND	ND	ND	ND	ND	ND
QrHDA19	OCV3_primec7262/OCV4_rep_c13389	44.2	66.6	36.3	56.6	341.1	28.5
QrHDA6	OCV4_rep_c26599	ND	ND	ND	ND	ND	ND
QrHDA9-like	OCV3_prime_rep_c68588	8.7	5.8	7.6	50.4	26.0	6.1
QrHDA9	OCV4_rep_c14185	39.4	63.2	31.2	37.0	39.1	11.2
QrHDA5	Loc_6160_Tr_2_9_Conf_0.625_Len_2930	20.2	24.1	40.5	25.7	31.2	24.4
QrHDA15	OCV4_rep_c12421	136.5	169.8	186.5	370.4	158.8	308.5
QrHDA14	OCV3_prime_c9769	14.4	56.6	31.2	56.6	354.1	186.3
QrHDA8	Loc_15145_Tr_8_9_Conf_0.280_Len_1833	275.9	428.6	419.4	254.1	742.1	523.4
QrHDA2	OCV4_rep_c15012	1747.4	1342.3	942.6	679.0	854.1	421.6
QrHDT1-like2	Loc_1428_Tr_2_6_Conf_0.650_Len_1466	369.1	422.7	449.8	256.2	225.2	353.3
QrHDT1-like1	Loc_8805_Tr_2_4_Conf_0.833_Len_1959	2822.9	3538.3	2427.0	3209.7	1852.7	3649.6
QrSRT1-like1	Loc_9090_Tr_3_7_Conf_0.611_Len_2531	0.0	0.0	0.0	1.0	0.0	0.0

QrSRT1-like2	Loc_26807_Tr_3_3_Conf_0.778_Len_1873	20.2	11.7	15.2	159.5	53.4	76.4
QrSRT2	OCV3_prime_c3720	300.8	327.9	318.1	163.6	962.2	251.5

Table S2. Normalized read counts of *Quercus suber* homologs of DNA and histone epigenetic modifiers as a result of the genome mapping of previously 454 sequenced libraries. The tissues analyzed include: acorns in 3 development stages (S2, S3/4, S5) [48], in embryos (E) [48], in good and bad quality cork (C.Q) [49], in first (1F and 1M) and last stages (2F and 2M) of female (F) and male flower (M) development [50], in roots (R.) with medium (MD), severe (SD) or without drought stress (Ww) [51], in samples with red and open buds (ROp) and in samples with dormant and swollen buds (D/Sw) [52].

		**Buds**		**Male Flowers**		**Female Flowers**		**Acorns**			**Embryos**	**Cork Quality**		**Roots**		
**Gene name**	**Locus tag**	**ROp**	**DSw**	**1M**	**2M**	**1F**	**2F**	**S2**	**S3S4**	**S5**		**Good**	**Bad**	**WW**	**MD**	**SD**
**DNA Methyltransferases**																
QsMET1	CFP56_38722	19.0	37.8	9.7	0.0	6.6	2.7	21.5	61.8	29.4	12.0	10.0	11.3	18.7	19.3	10.4
QsCMT1	CFP56_24702	24.8	2.8	16.1	0.0	5.5	6.7	0.0	0.0	10.2	8.7	0.0	0.9	12.5	15.1	11.7
QsCMT2.1	CFP56_06331	5.8	7.0	1.6	1.9	5.5	1.3	2.6	37.7	3.4	9.8	2.5	5.7	18.7	3.3	3.3
QsCMT3	CFP56_35720	67.2	71.4	4.8	0.0	5.5	5.4	35.2	32.6	29.4	14.9	6.7	0.9	29.1	30.6	9.1
QsDRM2	CFP56_35392	213.4	219.7	32.2	69.6	12.1	18.8	0.9	3.4	12.4	10.9	352.9	627.0	6.7	5.7	9.8
QsDRM3	CFP56_66797	2.9	8.4	1.6	5.8	18.8	21.5	26.6	37.7	6.8	29.0	3.3	0.9	20.0	7.5	19.5
QsDNMT2	CFP56_40985	16.1	30.8	14.5	15.5	15.4	2.7	8.6	5.1	7.9	23.6	6.7	11.3	19.6	10.4	5.2
QsCMT2.2	CFP56_59471	0.0	0.0	1.6	5.8	1.1	0.0	4.3	20.6	0.0	3.3	0.8	2.8	3.3	1.9	0.7
**DNA Demethylases**																
QsDME	CFP56_06273	23.4	15.4	19.3	29.0	30.9	20.1	94.5	272.8	40.7	35.9	10.0	17.9	52.0	54.2	29.3
QsROS1	CFP56_05353	16.1	15.4	66.0	5.8	39.7	26.9	209.6	332.8	369.8	117.4	51.6	30.2	166.8	199.2	168.1
QsDML2	CFP56_35285	0.0	0.0	0.0	0.0	0.0	0.0	0.0	0.0	0.0	0.0	0.0	0.0	0.0	0.0	0.0
**Histone Acetyltransferases**																
QsHAC1	CFP56_16103	21.9	16.8	4.8	21.3	5.5	12.1	128.0	168.1	109.7	57.3	48.3	45.3	63.2	83.8	73.6
QsHAC1-like1	CFP56_07357	0.0	0.0	4.8	0.0	0.0	0.0	0.9	0.0	11.3	13.4	0.0	0.0	0.8	2.8	2.0
QsHAC1-like2	CFP56_07360	0.0	0.0	0.0	0.0	0.0	0.0	0.0	0.0	0.0	0.0	0.0	0.0	0.0	0.0	0.0
QsGCN5	CFP56_29027	14.6	21.0	16.1	5.8	8.8	0.0	23.2	49.7	18.1	12.7	6.7	7.5	17.9	26.4	45.0
QsHAG2	CFP56_43084	51.2	33.6	29.0	3.9	58.5	30.9	30.1	37.7	6.8	50.4	8.3	17.9	10.8	8.5	27.4
QsHAM1	CFP56_58450	23.4	0.0	29.0	30.9	24.3	34.9	97.1	66.9	40.7	34.4	11.7	28.3	87.8	103.6	107.5
QsHAF1	CFP56_26298	5.8	15.4	11.3	1.9	4.4	8.1	118.5	145.8	95.0	66.7	14.2	21.7	69.1	46.6	104.9
QsELP3	CFP56_52694	52.6	35.0	32.2	38.6	24.3	24.2	25.8	10.3	57.7	36.6	25.0	49.0	34.1	28.7	30.0
**Histone Methyltransferases**																

QsSET40	CFP56_02401	0.0	0.0	12.9	25.1	3.3	6.7	4.3	0.0	20.4	15.6	5.0	2.8	3.3	3.8	2.0
QsASHR2	CFP56_23528	4.4	14.0	4.8	19.3	11.0	6.7	2.6	5.1	2.3	4.0	0.0	0.0	5.4	1.9	2.0
QsASHR2-like	CFP56_23526	4.4	7.0	24.1	36.7	28.7	13.4	6.0	8.6	9.0	9.8	1.7	0.0	3.3	1.4	6.5
QsSUVR5	CFP56_22604	20.5	18.2	6.4	5.8	13.2	13.4	45.5	63.5	58.8	25.4	10.8	6.6	41.2	44.7	100.3
QsCLF	CFP56_48440	13.2	11.2	19.3	13.5	8.8	1.3	39.5	30.9	22.6	17.4	10.0	3.8	59.9	33.0	61.2
QsATX3	CFP56_04889	10.2	9.8	0.0	15.5	24.3	13.4	39.5	128.7	26.0	13.8	6.7	6.6	53.2	54.2	34.5
QsSUVH6	CFP56_40587	0.0	2.8	3.2	13.5	2.2	2.7	7.7	15.4	18.1	13.4	4.2	4.7	7.9	8.5	2.0
QsSUVR2	CFP56_56207	10.2	35.0	16.1	11.6	22.1	16.1	55.0	42.9	65.6	48.2	24.1	6.6	29.5	41.4	24.1
QsATX2	CFP56_26820	17.5	9.8	6.4	3.9	5.5	1.3	28.3	85.8	30.5	27.5	14.2	3.8	12.5	16.0	55.4
ATXR2	CFP56_29061	0.0	12.6	0.0	0.0	1.1	0.0	8.6	72.1	15.8	3.6	1.7	0.0	20.4	17.4	3.3
QsSUVR3	CFP56_29051	0.0	1.4	3.2	11.6	1.1	4.0	0.0	17.2	2.3	2.2	3.3	0.9	2.9	0.0	2.6
QsSUVH9	CFP56_44980	5.8	15.4	6.4	13.5	12.1	5.4	16.3	30.9	18.1	12.3	1.7	4.7	14.6	17.4	13.0
QsASHH2	CFP56_13855	23.4	8.4	11.3	7.7	14.3	14.8	132.3	101.2	98.4	44.6	13.3	4.7	69.9	51.8	59.3
QsSUVR4	CFP56_60043	0.0	0.0	0.0	0.0	0.0	0.0	0.0	0.0	10.2	25.7	0.0	0.0	0.8	0.0	0.0
QsASHH3	CFP56_26485	20.5	2.8	16.1	17.4	28.7	12.1	4.3	30.9	20.4	43.5	25.0	14.1	25.4	18.8	20.2
QsATXR1	CFP56_15756	0.0	0.0	0.0	0.0	0.0	0.0	0.0	0.0	0.0	0.0	0.0	0.0	0.0	0.0	0.0
QsSUVH4-like	CFP56_01500	21.9	0.0	0.0	5.8	1.1	0.0	1.7	3.4	26.0	6.2	0.8	0.0	0.8	10.4	7.8
QsATXR6	CFP56_57230	13.2	16.8	12.9	0.0	0.0	2.7	0.0	0.0	0.0	2.9	0.0	0.0	0.8	2.8	4.6
QsASHR3	CFP56_53193	10.2	14.0	6.4	0.0	27.6	6.7	5.2	10.3	13.6	16.7	5.0	3.8	3.7	4.7	18.9
QsATXR7	CFP56_14674	2.9	1.4	8.0	0.0	9.9	6.7	17.2	20.6	9.0	18.5	20.8	4.7	9.6	12.2	32.6
QsATX5	CFP56_25529	16.1	18.2	0.0	11.6	5.5	2.7	50.7	46.3	53.2	15.9	5.0	8.5	36.6	72.5	50.8
QsSUVH1	CFP56_34169	13.2	9.8	16.1	9.7	4.4	12.1	34.4	25.7	39.6	16.7	12.5	16.0	26.2	24.5	20.2
QsSUVH3	CFP56_28232	19.0	2.8	9.7	3.9	16.5	4.0	46.4	29.2	45.2	18.5	12.5	15.1	23.7	26.8	20.2
QsASHH1	CFP56_54040	5.8	0.0	8.0	0.0	16.5	6.7	10.3	8.6	29.4	26.8	0.8	1.9	13.3	12.2	5.9
QsSUVH4	CFP56_37589	35.1	4.2	3.2	29.0	32.0	67.1	52.4	46.3	96.1	29.4	15.0	25.5	48.7	89.0	31.9
QSASHR1	CFP56_31286	0.0	9.8	24.1	17.4	7.7	10.7	30.9	0.0	1.1	18.8	4.2	8.5	17.5	26.4	11.1
QsATXR5	CFP56_53906	1.5	0.0	9.7	15.5	8.8	2.7	12.0	5.1	4.5	4.3	0.0	0.0	0.8	0.9	0.0
QsSWN	CFP56_32659	0.0	0.0	1.6	19.3	2.2	5.4	20.6	8.6	24.9	20.3	6.7	6.6	30.4	22.1	22.2
QsSET41	CFP56_40485	0.0	0.0	1.6	0.0	2.2	4.0	16.3	6.9	1.1	5.4	5.0	0.0	2.9	13.7	10.4
QsSUVH5b	CFP56_65722	0.0	0.0	0.0	0.0	0.0	0.0	0.0	0.0	0.0	0.0	0.0	0.0	0.0	0.0	0.0
QsSUVH5a	CFP56_65724	0.0	0.0	0.0	0.0	0.0	0.0	0.0	0.0	0.0	0.0	0.0	0.0	0.0	0.0	0.0
QsSUVR4-likeb	CFP56_76543	0.0	0.0	0.0	0.0	0.0	0.0	0.0	0.0	0.0	0.0	0.0	0.0	0.0	0.0	0.0
QsSUVR4-likea	CFP56_36057	0.0	0.0	0.0	0.0	0.0	0.0	0.0	0.0	0.0	0.0	0.0	0.0	0.0	0.0	0.0

QsSET10	CFP56_45284	21.9	0.0	11.3	9.7	2.2	0.0	3.4	13.7	15.8	10.1	10.0	2.8	3.3	14.6	14.3
QSATXR3	CFP56_27073	1.5	2.8	0.0	0.0	1.1	4.0	13.7	1.7	6.8	2.2	0.0	0.0	5.0	4.7	10.4
**Histone Demethylases**																
QsJMJ18	CFP56_59257	4.4	8.4	8.0	25.1	2.2	4.0	27.5	6.9	45.2	44.6	10.8	12.3	9.2	7.1	13.7
QsKDM3B-like	CFP56_10640	8.8	2.8	3.2	9.7	5.5	0.0	25.8	20.6	23.7	20.3	10.8	17.9	12.1	16.0	28.0
QsJMJ25-like1	CFP56_48908	10.2	7.0	20.9	42.5	13.2	18.8	42.1	41.2	49.8	46.4	15.8	0.9	35.4	26.4	26.7
QsJMJ25-like2	CFP56_47938	0.0	0.0	0.0	0.0	0.0	0.0	0.0	0.0	0.0	0.0	0.0	0.0	0.0	0.0	0.0
QsJMJ25-like3	CFP56_18193	5.8	12.6	9.7	0.0	7.7	2.7	42.1	27.4	40.7	15.9	10.8	13.2	18.7	28.3	11.1
QsJMJ25-like4	CFP56_18195	4.4	0.0	1.6	0.0	1.1	1.3	23.2	8.6	26.0	16.3	15.0	1.9	1.7	3.8	0.7
QsJMJ24	CFP56_37500	8.8	4.2	12.9	1.9	11.0	2.7	55.0	39.5	83.7	47.5	30.0	2.8	28.7	35.3	81.4
QsKDM3D-like	CFP56_34178	59.9	72.8	3.2	5.8	22.1	8.1	104.8	137.2	229.6	64.9	28.3	5.7	59.5	56.0	33.9
QsJMJD6Aa	CFP56_37891	17.5	4.2	4.8	11.6	3.3	5.4	0.0	0.0	1.1	6.5	5.0	5.7	2.9	5.7	9.1
QsJMJD6Ab	CFP56_50389	0.0	0.0	0.0	0.0	0.0	0.0	0.0	0.0	0.0	0.0	0.0	0.0	0.0	0.0	0.0
QsJMJ16	CFP56_76189	0.0	0.0	1.6	0.0	1.1	1.3	16.3	22.3	37.3	3.6	0.0	10.4	9.2	37.2	13.0
QsJMJ19-like	CFP56_17215	2.9	5.6	3.2	3.9	2.2	4.0	28.3	13.7	10.2	21.7	7.5	2.8	25.4	24.5	15.6
QsJMJ706	CFP56_36126	0.0	0.0	0.0	0.0	0.0	0.0	0.0	0.0	0.0	0.0	0.0	0.0	0.0	0.0	0.0
QsJMJ706-like	CFP56_37564	80.4	454.9	130.3	394.1	200.8	155.8	88.5	22.3	139.1	110.9	40.8	52.8	281.2	331.1	562.9
QsLDL3	CFP56_08883	5.8	0.0	4.8	5.8	2.2	5.4	36.1	60.0	35.1	13.0	2.5	11.3	18.7	23.1	23.5
QsELF6	CFP56_41197	7.3	0.0	1.6	0.0	1.1	1.3	28.3	13.7	15.8	8.0	0.8	7.5	9.2	7.1	8.5
QsJMJD6B	CFP56_61743	19.0	14.0	12.9	9.7	8.8	37.6	42.9	75.5	61.1	41.0	20.8	6.6	27.5	25.9	37.1
QsJMJ32-like	CFP56_26267	0.0	0.0	0.0	0.0	0.0	0.0	0.0	0.0	0.0	0.0	0.0	0.0	0.0	0.0	0.0
QsJMJ32	CFP56_26269	0.0	0.0	0.0	0.0	0.0	0.0	0.0	0.0	0.0	0.0	0.0	0.0	0.0	0.0	0.0
QsREF6	CFP56_69927	119.8	89.6	67.6	69.6	60.7	67.1	128.0	51.5	151.5	48.6	35.8	49.0	104.8	130.5	95.1
QsLDL2	CFP56_19117	1.5	0.0	0.0	0.0	1.1	0.0	1.7	5.1	3.4	4.7	0.8	1.9	5.8	15.5	2.0
QsLDL1	CFP56_55051	4.4	14.0	3.2	3.9	3.3	4.0	4.3	8.6	18.1	2.9	2.5	1.9	3.7	3.3	3.9
QsKDM5	CFP56_46886	0.0	7.0	0.0	0.0	7.7	6.7	82.5	30.9	58.8	15.2	3.3	1.9	13.7	16.0	18.2
QsJMJ30	CFP56_23213	1.5	2.8	1.6	0.0	5.5	0.0	8.6	15.4	5.7	4.7	15.0	5.7	4.2	8.0	9.8
QsFLD	CFP56_03400	0.0	5.6	4.8	3.9	0.0	0.0	9.4	22.3	28.3	4.0	3.3	1.9	12.5	15.5	9.8
QsJMJ20	CFP56_45215	0.0	1.4	3.2	7.7	12.1	1.3	0.0	37.7	6.8	12.7	0.0	0.9	18.3	5.7	18.2
**Histone Deacetylases**																
QsHDA2	CFP56_55621	0.0	5.6	22.5	59.9	24.3	38.9	98.8	49.7	15.8	42.4	40.0	10.4	8.7	27.8	48.2
QsHDA5	CFP56_01537	20.5	14.0	19.3	40.6	18.8	29.5	55.8	36.0	63.3	26.1	19.1	14.1	30.0	35.3	5.9
QsHDA6	CFP56_50628	2.9	57.4	17.7	36.7	36.4	18.8	39.5	30.9	54.3	44.6	32.5	35.8	17.9	21.7	31.9

QsHDA19-like2	CFP56_14676	39.5	46.2	49.9	42.5	67.3	94.0	67.0	41.2	36.2	54.0	40.0	39.6	38.3	53.2	62.5
QsHDA19	CFP56_26357	43.8	54.6	29.0	27.0	60.7	41.6	35.2	34.3	44.1	31.9	17.5	30.2	40.4	26.4	18.2
QsHDA9	CFP56_22884	20.5	46.2	48.3	65.7	51.8	53.7	55.8	30.9	74.6	79.7	13.3	111.3	113.1	100.3	61.9
QsHDA19-like1	CFP56_75988	0.0	0.0	0.0	0.0	0.0	0.0	0.0	0.0	0.0	0.0	0.0	0.0	0.0	0.0	0.0
QsHDA8	CFP56_03621	16.1	9.8	12.9	3.9	5.5	6.7	4.3	1.7	13.6	18.5	1.7	0.0	2.1	0.9	5.9
QsHDA14-like1	CFP56_64318	5.8	16.8	8.0	5.8	3.3	5.4	0.0	5.1	3.4	5.1	2.5	3.8	2.5	8.0	0.0
QsHDA14-like2	CFP56_32080	5.8	23.8	6.4	13.5	6.6	0.0	0.0	3.4	11.3	14.5	6.7	2.8	0.0	0.0	1.3
QsHDA14-like3	CFP56_65290	2.9	2.8	4.8	1.9	1.1	0.0	0.0	0.0	1.1	5.8	1.7	0.0	0.0	0.5	0.0
QsHDA15	CFP56_10834	67.2	89.6	54.7	23.2	75.0	83.2	137.4	87.5	80.3	105.1	124.0	61.3	74.9	105.0	89.3
QsHDT1-like1	CFP56_42573	371.2	340.1	194.7	156.5	142.3	111.4	78.2	126.9	47.5	58.0	100.7	216.9	131.9	119.6	138.1
QsHDT1-like3	CFP56_05349	49.7	63.0	74.0	40.6	46.3	102.0	48.1	48.0	64.5	88.1	70.8	138.6	43.3	16.0	39.7
QsSRT1-like3	CFP56_78126	2.9	4.2	3.2	25.1	3.3	4.0	8.6	6.9	5.7	21.7	0.8	5.7	10.0	2.8	3.3
QsSRT1-like5	CFP56_38596	5.8	0.0	0.0	3.9	0.0	4.0	0.9	1.7	6.8	6.9	1.7	0.0	1.7	0.0	0.0
QsSRT1-like4	CFP56_42400	4.4	0.0	0.0	1.9	0.0	6.7	1.7	6.9	0.0	12.7	0.0	2.8	0.0	0.0	2.6
QsSRT1-like2	-	ND	ND	ND	ND	ND	ND	ND	ND	ND	ND	ND	ND	ND	ND	ND
QsSRT1-like1	-	ND	ND	ND	ND	ND	ND	ND	ND	ND	ND	ND	ND	ND	ND	ND
QsSRT2	CFP56_42602	4.4	1.4	0.0	27.0	2.2	8.1	6.0	12.0	5.7	10.9	2.5	14.1	2.1	1.9	1.3
QsHDT1-like1	CFP56_51836	ND	ND	ND	ND	ND	ND	ND	ND	ND	ND	ND	ND	ND	ND	ND
QsHDA9-like	-	ND	ND	ND	ND	ND	ND	ND	ND	ND	ND	ND	ND	ND	ND	ND

Table S3. The identification of the loci encoding DNA and histone epigenetic modifier homologs of *Quercus suber*. Gene name, the correspondent locus tag, gene symbol and protein IDs (including splicing forms) are represented.

Gene Name	Locus Tag	Gene Symbol	Proteins
**DNA Methyltransferases**			
QsMET1	CFP56_38722	LOC111984282	XP_023871673.1
QsCMT1	CFP56_24702	LOC112002245	XP_023890169.1<br>XP_023890170.1
QsCMT2.1	CFP56_06331	LOC112001521	XP_023889464.1<br>XP_023889465.1
QsCMT3	CFP56_35720	LOC112031183	XP_023919638.1
QsDRM2	CFP56_35392	LOC112033986	XP_023922537.1
QsDRM3	CFP56_66797	LOC112032791	XP_023921322.1<br>XP_023921323.1

			XP_023921324.1<br>XP_023921321.1
QsDNMT2	CFP56_40985	LOC112003961	XP_023891955.1
QsCMT2.2	CFP56_59471	LOC112034184	XP_023922750.1
**DNA Demethylases**			
QsDME	CFP56_06273	LOC111983123	XP_023870550.1
QsROS1	CFP56_05353	LOC112039671	XP_023928322.1
QsDML2	CFP56_35285	LOC112020085	XP_023908412.1
**Histone Acetyltransferases**			
QsHAC1	CFP56_16103	LOC112034234	XP_023922811.1<br>XP_023922818.1
QsHAC1-like1	CFP56_07357	LOC111988497	XP_023876070.1
QsHAC1-like2	CFP56_07360	LOC111988316	XP_023875877.1
QsGCN5	CFP56_29027	LOC111998855	XP_023886737.1
QsHAG2	CFP56_43084	LOC112010794	XP_023898930.1
QsHAM1	CFP56_58450	LOC112012489	XP_023900637.1
QsHAF1	CFP56_26298	LOC111991837	XP_023879413.1<br>XP_023879414.1
QsELP3	CFP56_52694	LOC112000045	XP_023887919.1
**Histone Methyltransferases**			
QsSET40	CFP56_02401	LOC111984868	XP_023872255.1
QsASHR2	CFP56_23528	LOC111985014	XP_023872401.1
QsASHR2-like	CFP56_23526	LOC111984972	XP_023872355.1
QsSUVR5	CFP56_22604	LOC111985175	XP_023872585.1
QsCLF	CFP56_48440	LOC111988803	XP_023876366.1<br>XP_023876365.1
QsATX3	CFP56_04889	LOC111989353	XP_023876913.1
QsSUVH6	CFP56_40587	LOC111989703	XP_023877264.1<br>XP_023877266.1<br>XP_023877265.1
QsSUVR2	CFP56_56207	LOC111992201	XP_023879822.1<br>XP_023879817.1<br>XP_023879818.1

			XP_023879819.1<br>XP_023879820.1<br>XP_023879823.1
QsATX2	CFP56_26820	LOC111994171	XP_023881801.1<br>XP_023881799.1<br>XP_023881802.1<br>XP_023881800.1
QsATXR2	CFP56_29061	LOC111996077	XP_023883778.1
QsSUVR3	CFP56_29051	LOC111996085	XP_023883794.1
QsSUVH9	CFP56_44980	LOC111996522	XP_023884276.1<br>XP_023884277.1
QsASHH2	CFP56_13855	LOC111999241	XP_023887134.1<br>XP_023887133.1
QsSUVR4	CFP56_60043	LOC111999549	XP_023887448.1
QsASHH3	CFP56_26485	LOC112002855	XP_023890787.1<br>XP_023890783.1<br>XP_023890785.1<br>XP_023890784.1<br>XP_023890788.1
QsATXR1	CFP56_15756	LOC112005587	XP_023893650.1
QsSUVH4-like	CFP56_01500	LOC112036034	XP_023924619.1
QsATXR6	CFP56_57230	LOC112008849	XP_023896969.1
QsASHR3	CFP56_53193	LOC112009409	XP_023897510.1
QsATXR7	CFP56_14674	LOC112011828	XP_023899934.1<br>XP_023899935.1
QsATX5	CFP56_25529	LOC112017971	XP_023906233.1<br>XP_023906232.1
QsSUVH1	CFP56_34169	LOC112020905	XP_023909243.1
QsSUVH3	CFP56_28232	LOC112025017	XP_023913440.1
QsASHH1	CFP56_54040	LOC112035360	XP_023923957.1<br>XP_023923958.1<br>XP_023923956.1<br>XP_023923959.1
QsSUVH4	CFP56_37589	LOC112035570	XP_023924166.1

			XP_023924168.1<br>XP_023924167.1
QSASHR1	CFP56_31286	LOC112037474	XP_023926081.1
QsATXR5	CFP56_53906	LOC112038577	XP_023927165.1
QsSWN	CFP56_32659	LOC112038731	XP_023927343.1
QsSET41	CFP56_40485	LOC112039440	XP_023928085.1<br>XP_023928086.1
QsSUVH5b	CFP56_65722	LOC112015057	XP_023903188.1
QsSUVH5a	CFP56_65724	LOC112015060	XP_023903190.1
QsSUVR4-likeb	CFP56_76543	LOC112015520	XP_023903694.1
QsSUVR4-likea	CFP56_36057	LOC112032443	XP_023920971.1
QsSET10	CFP56_45284	LOC112020643	XP_023908960.1
QSATXR3	CFP56_27073	-	QSP042949.0
**Histone Demethylases**			
QsJMJ18	CFP56_59257	LOC111985798	XP_023873223.1<br>XP_023873225.1<br>XP_023873224.1
QsKDM3B-like	CFP56_10640	LOC111989488	XP_023877041.1<br>XP_023877042.1<br>XP_023877040.1
QsJMJ25-like1	CFP56_48908	LOC112003991	XP_023891992.1<br>XP_023891991.1<br>XP_023891990.1
QsJMJ25-like2	CFP56_47938	LOC112018887	XP_023907188.1
QsJMJ25-like3	CFP56_18193	LOC112030617	XP_023919056.1
QsJMJ25-like4	CFP56_18195	LOC112030626	XP_023919066.1<br>XP_023919065.1
QsJMJ24	CFP56_37500	LOC111995675	XP_023883369.1<br>XP_023883368.1<br>XP_023883365.1<br>XP_023883367.1
QsKDM3D-like	CFP56_34178	LOC112020902	XP_023909239.1
QsJMJD6Aa	CFP56_37891	LOC111991092	XP_023878641.1

QsJMJD6Ab	CFP56_50389	LOC112013333	XP_023901496.1
QsJMJ16	CFP56_76189	LOC112004294	XP_023892291.1<br>XP_023892289.1<br>XP_023892290.1
QsJMJ19-like	CFP56_17215	LOC112019264	XP_023907567.1<br>XP_023907566.1
QsJMJ706	CFP56_36127/6	LOC112010540	XP_023898652.1<br>XP_023898654.1<br>XP_023898653.1
QsJMJ706-like	CFP56_37564	LOC112030921	XP_023919361.1
QsLDL3	CFP56_08883	LOC112022596	XP_023910985.1
QsELF6	CFP56_41197	LOC112025120	XP_023913552.1
QsJMJD6B	CFP56_61743	LOC112026327	XP_023914775.1<br>XP_023914776.1
QsJMJ32-like	CFP56_26267	LOC112034921	XP_023923513.1
QsJMJ32	CFP56_26269	LOC112034640	XP_023923230.1
QsREF6	CFP56_69927	LOC112029048	XP_023917830.1
QsLDL2	CFP56_19117	LOC112032113	XP_023920630.1<br>XP_023920629.1
QsLDL1	CFP56_55051	LOC112033571	XP_023922114.1
QsKDM5	CFP56_46886	LOC112036450	XP_023925013.1
QsJMJ30	CFP56_23213	LOC112002893	XP_023890817.1
QsFLD	CFP56_03400	LOC112023717	XP_023912111.1
QsJMJ20	CFP56_45215	LOC111991489	XP_023879031.1
**Histone Deacetylases**			
QsHDA2	CFP56_55621	LOC112000524	XP_023888449.1
QsHDA5	CFP56_01537	LOC112036007	XP_023924594.1
QsHDA6	CFP56_50628	LOC112035676	XP_023924274.1
QsHDA19-like2	CFP56_14676	LOC112011819	XP_023899923.1<br>XP_023899924.1<br>XP_023899925.1
QsHDA19	CFP56_26357	LOC112003171	XP_023891130.1<br>XP_023891137.1

QsHDA9	CFP56_22884	LOC111996728	XP_023884497.1<br>XP_023884496.1
QsHDA19-like1	CFP56_75988	LOC112009697	XP_023897791.1
QsHDA8	CFP56_03621	LOC112005173	XP_023893191.1
QsHDA14-like1	CFP56_64318	LOC112012023	XP_023900151.1<br>XP_023900150.1<br>XP_023900153.1<br>XP_023900152.1
QsHDA14-like2	CFP56_32080	LOC112004322	XP_023892317.1
QsHDA14-like3	CFP56_65290	LOC112011809	XP_023899914.1
QsHDA15	CFP56_10834	LOC112024699	XP_023913091.1<br>XP_023913093.1<br>XP_023913092.1
QsHDT1-like1	CFP56_42573	LOC112002966	XP_023890901.1
QsHDT1-like3	CFP56_05349	LOC112039702	XP_023928367.1
QsSRT1-like3	CFP56_78126	LOC112020807	XP_023909132.1
QsSRT1-like5	CFP56_38596	LOC112028975	XP_023917433.1
QsSRT1-like4	CFP56_42400	LOC112017795	XP_023906010.1<br>XP_023906011.1
QsSRT1-like2	-	LOC112025841	XP_023914295.1
QsSRT1-like1	-	LOC112027314	XP_023915762.1
QsSRT2	CFP56_42602	LOC112032808	XP_023921347.1<br>XP_023921348.1
QsHDT1-like1	CFP56_51836	LOC112035297	XP_023923893.1
QsHDA9-like	-	LOC112031837	XP_023920316.1
